# Energy Dependence of Measured CT Numbers on Substituted Materials Used for CT Number Calibration of Radiotherapy Treatment Planning Systems

**DOI:** 10.1371/journal.pone.0158828

**Published:** 2016-07-08

**Authors:** Reza Mahmoudi, Nasrollah Jabbari, Mehdi aghdasi, Hamid Reza Khalkhali

**Affiliations:** 1 Department of Medical Physics and Imaging, Urmia University of Medical Sciences, Urmia, Iran; 2 Solid Tumor Research center, Urmia University of Medical Sciences, Urmia, Iran; 3 Radiotherapy Center of the Omid Hospital, Urmia, Iran; 4 Radiotherapy Center of Parto, Urmia, Iran; 5 Department of Biostatistics and Epidemiology, Urmia University of Medical Sciences, Urmia, Iran; North Shore Long Island Jewish Health System, UNITED STATES

## Abstract

**Introduction:**

For accurate dose calculations, it is necessary to provide a correct relationship between the CT numbers and electron density in radiotherapy treatment planning systems (TPSs). The purpose of this study was to investigate the energy dependence of measured CT numbers on substituted materials used for CT number calibration of radiotherapy TPSs and the resulting errors in the treatment planning calculation doses.

**Materials and Methods:**

In this study, we designed a cylindrical water phantom with different materials used as tissue equivalent materials for the simulation of tissues and obtaining the related CT numbers. For evaluating the effect of CT number variations of substituted materials due to energy changing of scanner (kVp) on the dose calculation of TPS, the slices of the scanned phantom at three kVp's were imported into the desired TPSs (MIRS and CorePLAN). Dose calculations were performed on two TPSs.

**Results:**

The mean absolute percentage differences between the CT numbers of CT scanner and two treatment planning systems for all the samples were 3.22%±2.57% for CorePLAN and 2.88%±2.11% for MIRS. It was also found that the maximum absolute percentage difference between all of the calculated doses from each photon beam of linac (6 and 15 MV) at three kVp's was less than 1.2%.

**Discussion:**

The present study revealed that, for the materials with effective low atomic number, the mean CT number increased with increasing energy, which was opposite for the materials with an effective high atomic number. We concluded that the tissue substitute materials had a different behavior in the energy ranges from 80 to 130 kVp. So, it is necessary to consider the energy dependence of the substitute materials used for the measurement or calibration of CT number for radiotherapy treatment planning systems.

## Introduction

Cancer is a major public health issue in most of the countries. There are multiple external and internal factors that may act together to cause cancer. Some of the cancer risk factors such as environmental factors can be avoided, while the others like inherited genetic mutations and aging cannot be avoided. The world population is getting older and the effect of environmental factors such as carcinogenic elements is the most important factor for causing cancer [1].Radiation therapy has an important role in cancer treatment. It has been shown that about 50% of all cancer patients are candidates for radiotherapy during their course of disease [2]. Radiation therapy is dependent on specialized equipment, so its percentage varies in different countries and regions [2]. In radiotherapy centers, dose distributions of planning target volumes (PTVs) and critical organs are assessed using appropriate treatment planning systems and the optimal technique is selected for each patient [3]. In radiotherapy, accurate dose calculation is possible only when the accurate data are obtained from the patient. These data include body contour, shape and density of the internal organs, location and spread of tumor volume, and so on. The best way to obtain these data is to use the three-dimensional imaging systems, including computed tomography (CT), magnetic resonance imaging (MRI), positron emission tomography (PET), and so on.

In radiotherapy treatment planning systems (TPSs), dose calculations are performed on the data of CT images. These images are imported into the treatment planning systems as the input data in the treatment planning process. To obtain the accurate delivery of radiotherapy, the accurate delineation of the treatment target is essential [4]. The CT scan images are used for contouring different treatment target volumes and normal surrounding tissues or organs at risk (OAR). In addition, CT images contain the quantitative data (CT values) which can be used to correct tissue heterogeneity in radiotherapy treatment plans. For accurate dose calculations, it is necessary to provide a correct relationship between the CT numbers expressed in Hounsfield units (HU) and electron density in treatment planning systems. The CT number values represent tissue electron densities and are directly related to the linear attenuation coefficients of tissues in the photon beam path length [5, 6].

It is clear that the CT number depends on several factors such as photon energy spectrum, detector sensitivity, geometrical configuration of the phantom system, and possibly reconstruction algorithm [7]. Previous studies have shown that different scanners with different energies may provide different CT number data [5,8].There are typically phantoms which are supplied by the manufacturers that can be used for scanner calibration and monitoring of scanner performance. When these CT calibration phantoms are used for monthly testing, the CT number tolerance of water is ±5 HU. Monthly tests are also done with materials that have different densities [9].

One of the best ways to ensure the accuracy of CT numbers used in radiotherapy is to verify the accuracy of data transformation from the CT scanner to treatment planning system or converting CT numbers to relative electron densities. The most common data verification method is the CT number calibration technique using tissue equivalent materials. However, the elemental composition of substitute materials differs from that of real tissues. So, the calibration curves of CT number to electron density can be significantly different from the real situation [10].

The use of tissue equivalent materials for the calibration procedure in the low-energy range of CT scanners may introduce inaccuracies in the dose calculations of treatment planning systems using high-energy photon beams [10]. Therefore, the aims of this study were to:

1. Determine whether CT numbers of substitute materials on the CT images of the same material vary on the scans done at different energies.
2. Evaluate the transfer accuracy of the CT numbers of substitute materials from different kVps of the CT scanner to different treatment planning systems.
3. Investigate the energy dependence of the measured CT numbers on the substituted materials used for the CT number calibration of radiotherapy treatment planning systems.
4. Determine the influence of variations in the CT numbers from different kVps on radiation dose calculations in the treatment planning systems using different therapeutic photon beam energies (6 and 15 MV).

## Material and Methods

### 1. Designed phantom

**Fig 1:**
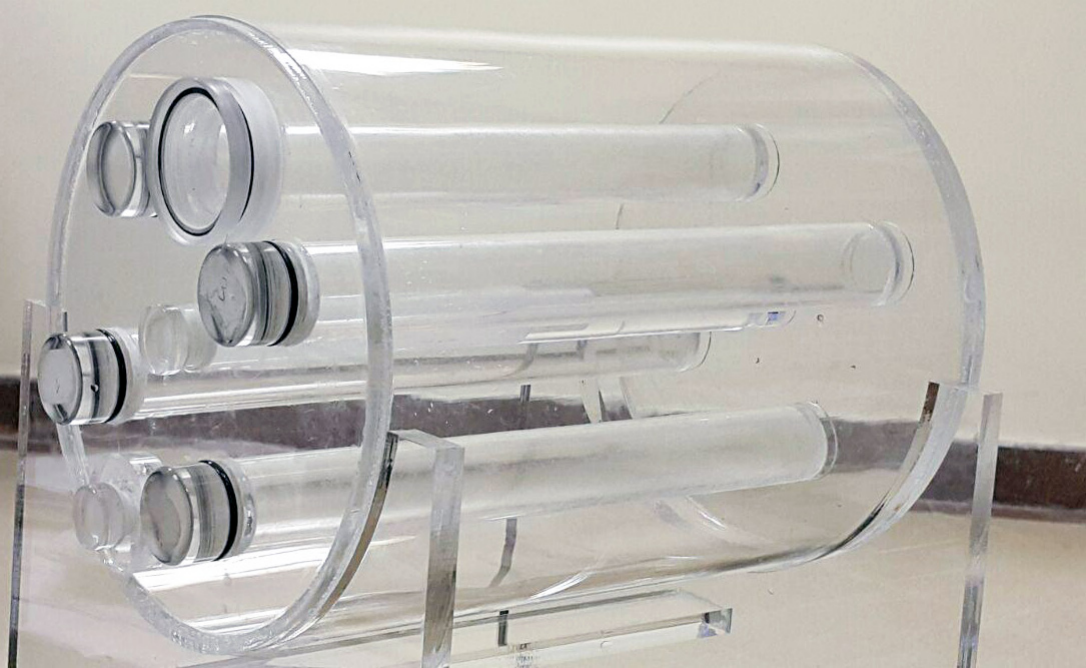
Designed phantom used in this study.

In this study, we designed a cylindrical water phantom made of Plexiglas with the length of 25 cm and diameter of 20 cm, which had 4 cylindrical tubes with 24 mm inner diameter and equal distance from the central axis of the phantom. The cylindrical tubes used in the phantom were designed to allow the insertion of different solids and liquid materials as tissue-equivalent materials. In addition, a cylindrical tube was designed at the center of the phantom to allow the insertion of ionization chamber for the experimental dose measurement. Fig 1 shows the designed phantom used in this study.

### 2. Treatment planning systems

In this study, two treatment planning systems (CorePLAN and MIRS) were used. CorePLAN, version 3.5.0.5 (Seoul C &J,Inc), is a commercial treatment planning system for three-dimensional conformal radiation therapy (3D-CRT) photon and electron beam dose calculations. CorePLAN can provide dose calculations based on collapsed cone convolution (CCC) and equivalent tissue air ratio (ETAR) algorithms for photon and Hogstrom algorithm for electron beams. The accuracy of its algorithms has been confirmed through numerous clinical tests [11–13]. MIRS (module integrated radiotherapy system) is a powerful modular high-technology system that allows three-dimensional planning for several radiation treatment modalities such as 3D-CRT, electron beam radiation therapy (EBRT), radiosurgery, and intensity modulated radiation therapy (IMRT) [14].

### 3. Tissue substitute materials

Different materials were used as tissue equivalent materials for the simulation of tissues and obtaining the related CT numbers. These materials include PVC as cancellous bone, polyethylene as fat, acrylic, and water as soft tissue, rice powder as muscle, aluminum as dense bone, and calf bone as a real cortical bone. The calf bone was purchased from a local butcher store. Geometry of tissue substitutes is in the form of a cylindrical tube and coaxial with central axis with 25 cm length and 24 mm inner diameter.

### 4. Acquisition of computed tomography and measurement of CT number

Scanning of the phantom was performed using a multislice CT scanner (SOMATOM Emotion 6; Siemens Healthcare, Germany). The manufacturer-supplied water phantom was scanned to verify the calibration of the CT scanner. Then, the designed phantom with tissue substitute materials was placed on top of the CT scanner table and acquisition was performed at three x- ray energies (80, 110, and 130 kVp) using brain scanning protocol with a fixed mAs established at 230 for three kVp’s.

The slice thickness for each scan was 5 mm. After a scan was completed, the substitute material's CT numbers at each kVp was measured at the scanner console. Each pixel within the region-of-interest (ROI) on the CT images had one associated value (CT number). For each kVp, the mean CT number was determined in a slice at the circular ROI with the area of approximately 2 cm^2^. This process was performed on the five slices at the center of the phantom and the mean and standard deviation (SD) of the CT numbers were recorded for each tissue substitute material.

After the acquisition of the CT, the CT images were imported into the radiotherapy planning systems using a DICOM format. For each tissue material, the mean and standard deviation of the CT numbers were also obtained on the treatment planning systems, similar to the scanner console.

Finally, for each kVp, three series of the CT numbers were recorded for each substitute material; the first series was obtained from the scanner, the second from CorePLAN, and the third from MIRS treatment planning system.

### 5. Medical linear accelerator

All the experimental measurements were performed on a Siemens PRIMUS medical linear accelerator. Siemens PRIMUS is a dual photon standing-wave linac. Microwave excitation may be induced by either klystron or magnetron. The triode (gridded) gun is used for electron injection. The bending magnet system is a 270° loop with fixed slits. In this study, two photon beams of linac (6 and 15 MV) were investigated.

### 6. Dose calculations using treatment planning systems

For evaluating the effect of CT number variations of the substituted materials due to the energy changing of scanner (kVp) during CT-scan acquisition on the dose calculation of treatment planning systems, the slices of the scanned phantom at three kVp's were imported into the desired treatment planning systems (MIRS and CorePLAN). Dose calculations were performed on two treatment planning systems at the source to skin distance (SSD) of 100 cm for the 6 and 15 MV photon beams with the field size of 10×10 cm^2^. Accordingly, the planning target volumes (PTVs) were selected at the center of the designed phantom and irradiated with 100 MU.

### 7. Statistical methods

For comparing the CT number variation of each substitute material with energy changes, the statistical paired t-test was used. In addition, we used paired t-test to prove there was no systematic difference between the CT numbers of the scanner and two treatment planning systems and also we used the relative percentage difference as a method to prove the similarity between the CT numbers of the scanner and two TPSs [15]. P-values of less than 0.05 were considered statistically significant.

## Results

The actual values of data are provided in Tables 1 and 2, which contain mean and standard deviation of the CT numbers for different materials at three kVps, results of paired t-test (p-values), and absolute percentage differences between the CT numbers of each substitute material obtained from the scanner and two TPSs at each kVp.

**Table 1:** Results of mean and standard deviation (M±SD) CT numbers, paired t-test results, and absolute percentage differences between the CT numbers of each substitute material obtained from the scanner and CorePLAN treatment planning system at each energy (kVp).

Substituted materials	Energy of CT-scanner (kVp)	Scanner CT- numbers (M±SD)	CorePLAN CT- numbers (M±SD)	P-values (paired t-test)	Absolute difference (%)
Acrylic	80	101 ± 4.01	105.36 ± 11.39	0.40	4.22
	110	123.26 ± 2.44	121.80± 7.99	0.22	1.19
	130	128 ± 2.25	126.32 ± 7.21	0.35	1.32
Al	80	3069.6 ± 0.56	3074.6± 0.98	0.76	0.16
	110	3065.4 ± 5.03	3070 ± 1.80	0.26	0.15
	130	2858.04 ± 172.28	2936.76 ± 37.2	0.76	2.71
Bone	80	2083.6 ± 111.01	2217 ± 62.58	0.35	6.20
	110	1607.04 ± 191.95	1716.88 ± 54.55	0.02	6.60
	130	1471.84 ± 106.46	1571.4 ± 34.20	0.29	6.54
Polyethylene	80	-109.76 ± 2.69	-109.61 ± 13.25	0.85	0.13
	110	-82.16 ± 2.01	-83.86 ± 10.01	0.23	2.04
	130	-75.67 ± 1.31	-72.20± 6.26	0.58	4.69
PVC	80	1304.4 ± 13.46	1306.36 ± 11.39	0.58	0.15
	110	954.31 ± 11.38	960.46 ± 14.87	0.64	0.64
	130	830.06 ± 8.10	785.44 ± 12.52	0.99	5.57
Rice powder	80	-110.24 ± 3.21	-108.30± 9.37	0.29	1.17
	110	-93.28 ± 3.12	-87.23± 8.01	0.81	6.70
	130	-76.36 ± 2.07	-73.32 ± 5.63	0.97	4.06
Water	80	1.02 ± 0.40	0.96±0.30	0.75	6.06
	110	1.44 ± 0.60	1.35± 0.50	0.40	6.45
	130	1.67 ± 0.50	1.76 ± 0.60	0.29	5.24

Absolute differences (%): Range = 0.13–6.70 Mean = 3.42 SD = 2.57.

**Table 2:** Results of mean and standard deviation (M±SD) CT numbers, paired t-test results, and absolute percentage differences between the CT numbers of each substitute material obtained from the scanner and MIRS treatment planning system at each energy (kVp).

Substituted materials	Energy of CT-scanner (kVp)	Scanner CT- numbers (M±SD)	CorePLAN CT- numbers (M±SD)	P-values (paired t-test)	Absolute difference (%)
Acrylic	80	101 ± 4.01	105.40 ± 22.7	0.67	4.26
	110	123.26 ± 2.44	121.45 ± 17.31	0.76	1.45
	130	128 ± 2.25	131.14 ± 10.65	0.43	1.65
Al	80	3069.6 ± 0.56	3069.97 ± 1.61	0.26	0.012
	110	3065.4 ± 5.03	3069.84 ± 1.28	0.52	0.14
	130	2858.04 ± 172.28	2891.92 ± 24.95	0.62	1.17
Bone	80	2083.6 ± 111.01	2142.22 ± 72.49	0.14	2.77
	110	1607.04 ± 191.95	1686.14 ± 43.81	0.49	4.80
	130	1471.84 ± 106.46	1569.48 ± 32.51	0.12	6.42
Polyethylene	80	-109.76 ± 2.69	-108.60 ± 16.41	0.87	1.06
	110	-82.16 ± 2.01	-85.05 ± 8.68	0.91	3.45
	130	-75.67 ± 1.31	-73.96 ± 7.40	0.05	2.28
PVC	80	1304.4 ± 13.46	1291.08 ± 21.42	0.87	1.02
	110	954.31 ± 11.38	942.70 ± 19.08	0.91	1.22
	130	830.06 ± 8.10	825.96 ± 10.55	0.05	0.49
Rice powder	80	-110.24 ± 3.21	-105.76 ± 11.38	0.47	4.14
	110	-93.28 ± 3.12	-87.16 ± 10.44	0.71	6.78
	130	-76.36 ± 2.07	-78.72 ± 6.68	0.80	3.04
Water	80	1.02 ± 0.40	0.96 ± 0.30	0.41	6.06
	110	1.44 ± 0.60	1.52 ± 0.60	0.65	5.40
	130	1.67 ± 0.50	1.72 ± 0.50	0.73	2.94

Absolute differences (%): Range = 0.012–6.78 Mean = 2.88 SD = 2.11.

Results of the paired t-test showed that no significant difference existed between the CT numbers of the scanner and two TPSs for all the materials at three kVp's (p-value > 0.05); therefore, there was no evidence of systematic difference. In addition, the absolute percentage differences between the CT numbers of CT scanner and two TPSs at three kVps of the scanner for all the materials used in this study were in the range of 0.012% to 6.78% in the Hounsfield unit values. Furthermore, the mean absolute percentage differences between the CT numbers of CT scanner and two treatment planning systems for all the samples were 3.22%±2.57% for CorePLAN and 2.88%±2.11% for MIRS. The deviation between CT numbers for all the test cases was within the recommend tolerance levels [16, 17].

**Fig 2:**
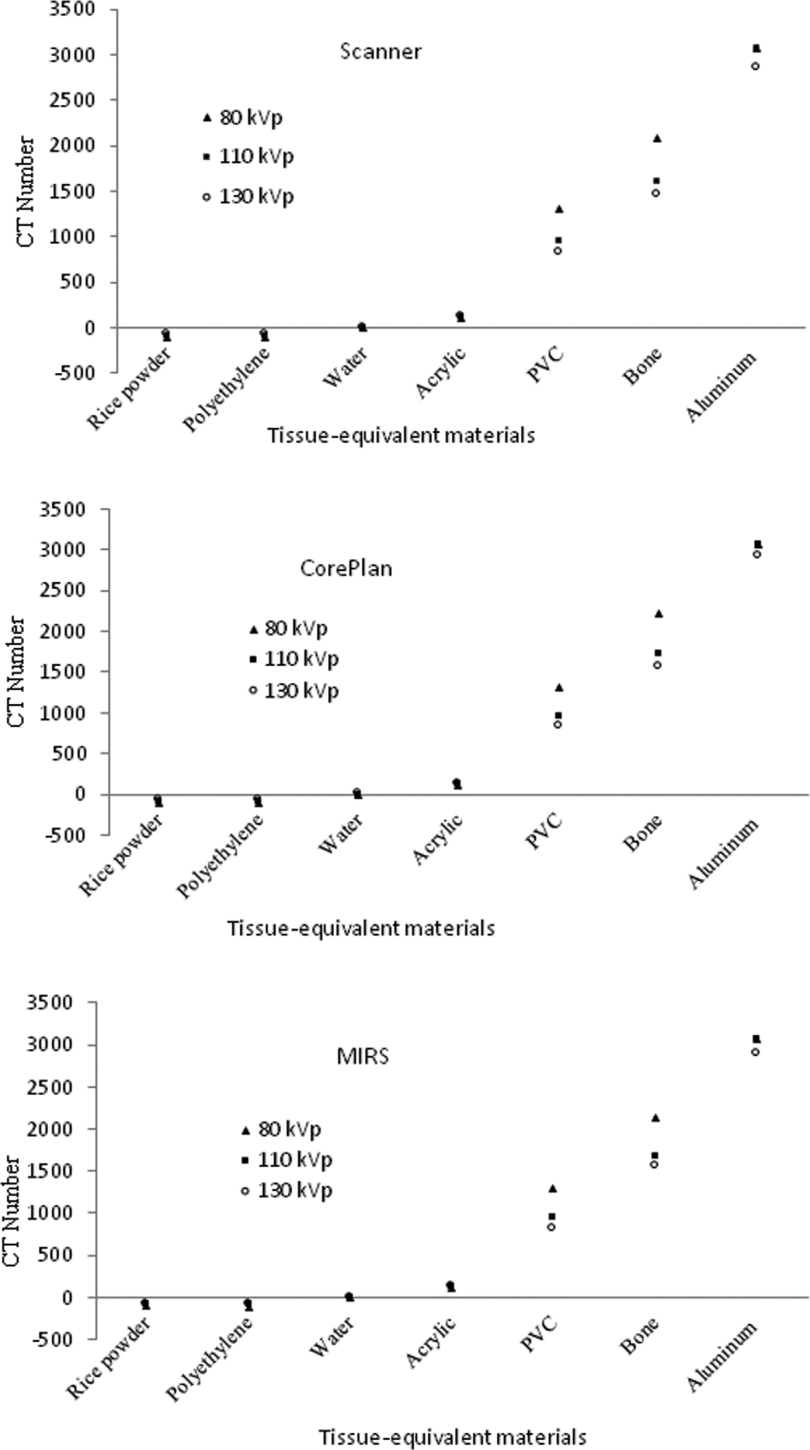
Variations in mean CT numbers among different energies of scanner (kVp) at the scanner and two treatment planning systems (CorePLAN and MIRS) for various tissue-equivalent materials.

The variations in mean CT numbers among different energies of scanner (kVp) at the scanner and two treatment planning systems (CorePLAN and MIRS) for various tissue-equivalent materials are shown in Fig 2.

**Fig 3:**
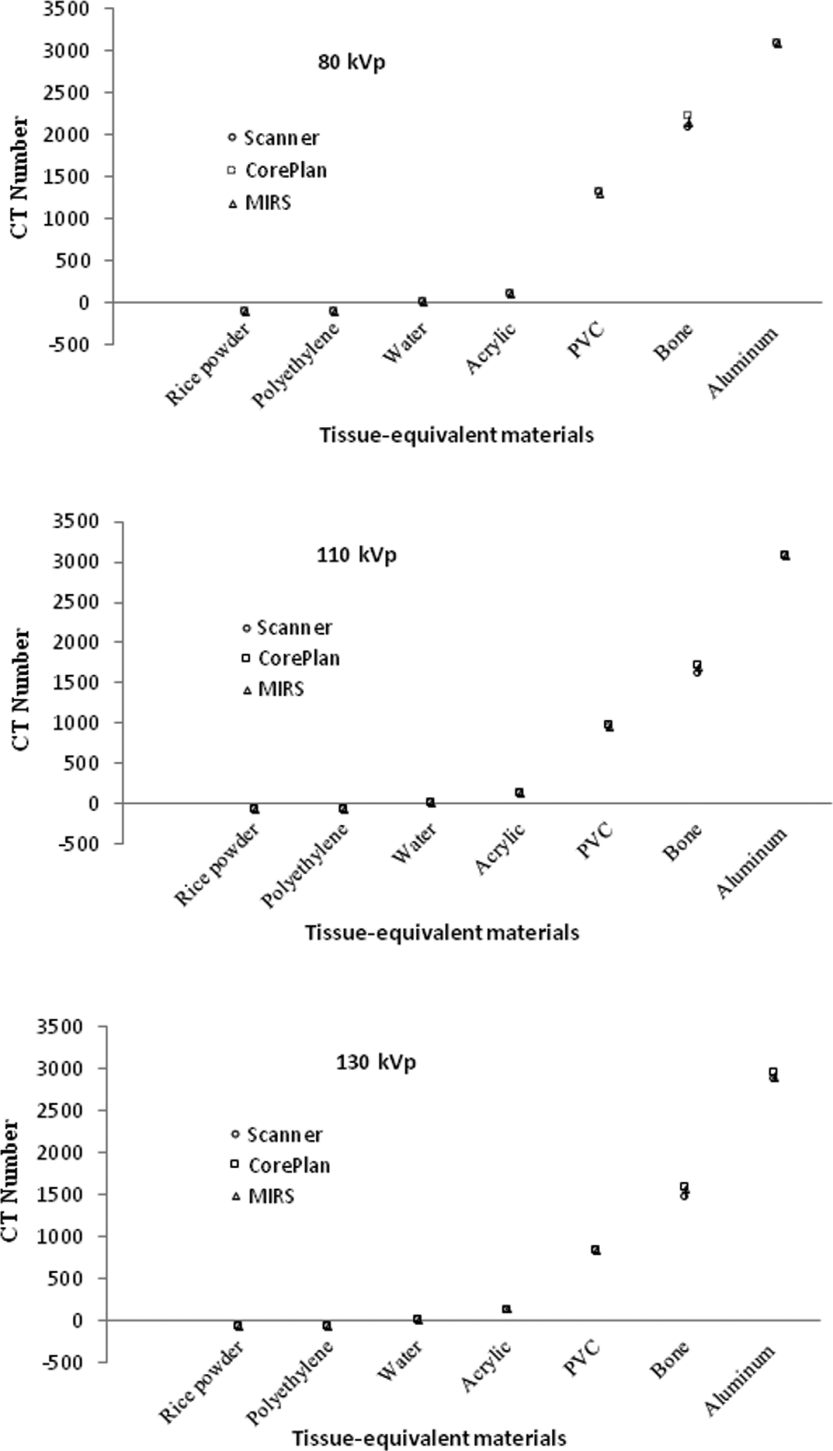
Mean CT number values of CT scanner, CorePLAN, and MIRS treatment planning systems at the same energy (kVp) for various tissue-equivalent materials.

The mean CT number values of the CT scanner, CorePLAN, and MIRS treatment planning systems at the same energy (kVp) for various tissue-equivalent materials are shown in Fig 3.

The results demonstrated that, there was a good similarity between the CT number values of the scanner and two treatment planning systems for the materials with effective low atomic number (rice powder, polyethylene, water, and acrylic) at three kVp's. However, there was a poor similarity between the CT numbers of the materials with effective high atomic number (PVC, bone, and aluminum) at three kVp's (Fig 2). In addition, the results indicated a good similarity between the CT numbers of the scanner and two treatment planning systems for all the materials at the same energy (Fig 3).

**Fig 4:**
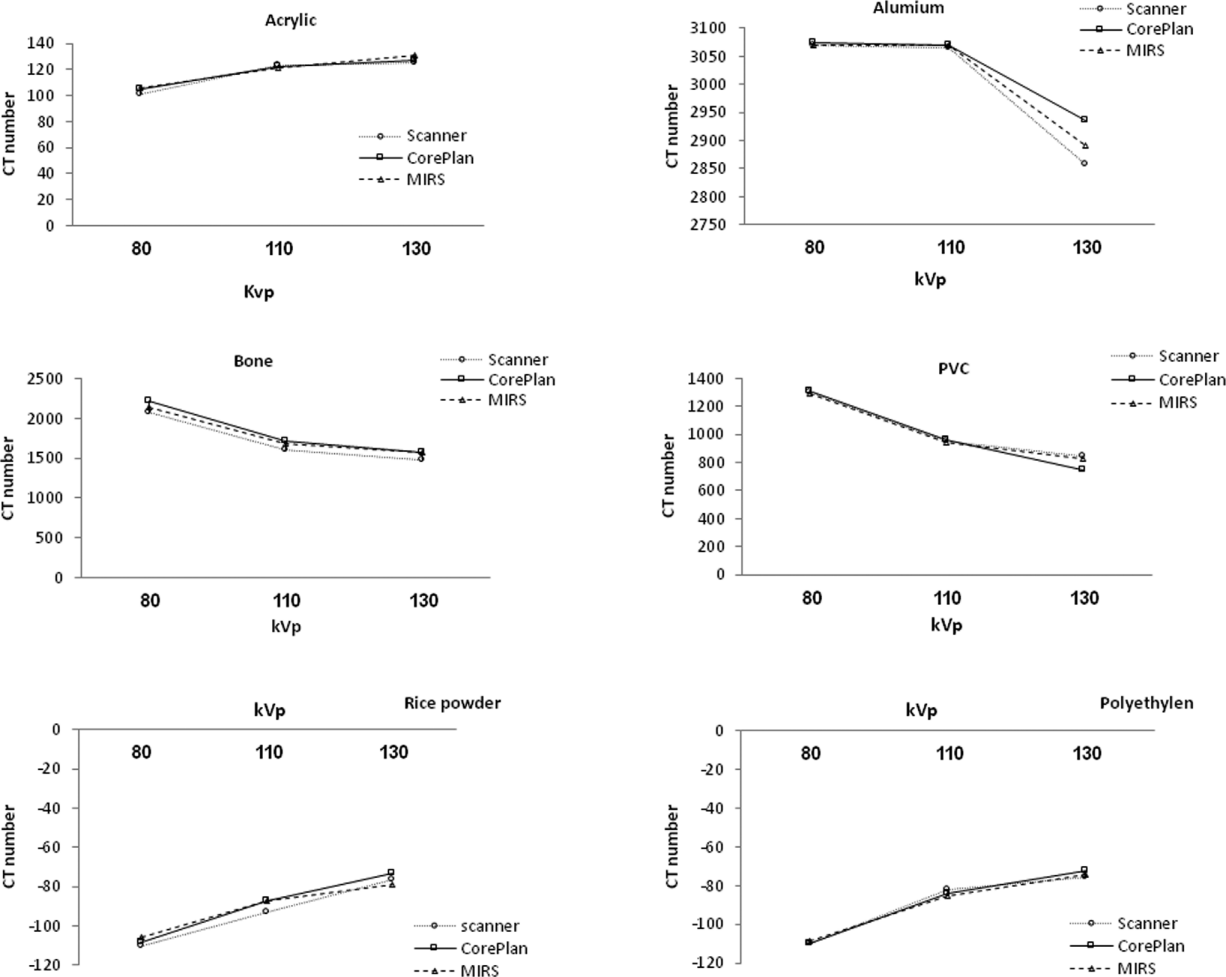
CT number variations according to change of kVp for different materials.

According to change of kVp for different materials, the CT number variations are shown in Fig 4. it was found that, for the materials with effective low atomic number, the mean CT number increased with increasing energy, which was opposite for the materials with an effective high atomic number.

The p-values resulted from the CT number variations of each substitute material with changing energy by statistical paired t–test are shown in Table 3.

**Table 3:** P-values resulted from the CT number variations of each substitute material with changing energy.

	CT-scanner			CorePlan			MIRS		
Materials	Energy (kVp)			Energy (kVp)			Energy (kVp)		
	80–110	110–130	80–130	80–110	110–130	80–130	80–110	110–130	80–130
Aluminum	0.115	0.232	0.874	0.332	0.082	0.452	0.993	0.359	0.315
Acrylic	0.797	0.611	0.369	0.972	0.713	0.462	0.697	0.861	0.779
Bone	0.014	0.004	0.007	0.062	0.544	0.975	0.449	0.562	0.658
Polyethylene	0.629	0.854	0.141	0.753	0.87	0.452	0.145	0.246	0.037
Rice powder	0.253	0.417	0.173	0.696	0.876	0.233	0.746	0.520	0.15
PVC	0.559	0.534	0.295	0.552	0.775	0.466	0.454	0.484	0.015
Water	0.029	0.206	0.173	0.706	0.786	0.056	0.626	0.33	0.089

The calculated doses of CorePLAN and MIRS treatment planning systems on the center of the scanned phantom at three kVp's of scanner using 6 and 15 MV photon beams of linac are shown in Tables 4 and 5, respectively.

**Table 4:** Calculated doses of CorePLAN on the center of the scanned phantom at three kVp's of scanner using 6 and 15 MV photon beams.

Calculated dose (cGy)						
Photon beam energy of Linac	6 MV			15 MV		
	kVp			kVp		
Material	80	110	130	80	110	130
Rice-powder	67.70	67.50	67.40	76.60	76.80	76.50
Polyethylene	67.90	67.70	67.80	76.70	76.90	76.60
Water	67.80	67.60	67.50	76.70	76.80	76.60
Acrylic	67.60	67.30	67.40	76.60	76.80	76.70
PVC	67.60	67.60	67.50	76.60	76.70	76.50
Bone	67.80	67.40	67.80	76.80	76.70	76.80
Aluminum	66.90	67.30	67.10	76.30	76.70	76.20

**Table 5:** Calculated doses of MIRS on the center of the scanned phantom at three kVp's of scanner using 6 and 15 MV photon beams.

Calculated dose (cGy)						
Photon beam energy of Linac	6 MV			15 MV		
	kVp			kVp		
Material	80	110	130	80	110	130
Rice-powder	66.60	66.50	66.30	77.50	77.30	77.20
Polyethylene	66.70	66.60	66.50	77.60	77.40	77.30
Water	66.60	66.60	66.50	77.60	77.60	77.40
Acrylic	66.50	66.40	66.40	77.70	77.60	77.40
PVC	66.50	66.50	66.40	77.50	77.40	77.30
Bone	66.70	66.50	66.30	77.70	77.70	77.70
Aluminum	66.60	66.80	67.20	77.50	77.70	78.40

It can be inferred from the analysis that the maximum absolute percentage difference between all of the calculated doses from each photon beam of linac (6 and 15 MV) at three kVp's was less than 1.2% (Tables 4 and 5).

## Discussion

In our paper, the focus of attention was on the variations of the substituted materials' CT number with x-ray energy in CT scanner and two treatment planning systems (CorePLAN and MIRS) and evaluating the effect of CT number variation on the dose calculations of photon beams. Our results described the influence of kVp on the CT number and variations associated with different substituted materials in the CT number calibration of radiotherapy treatment planning systems.

We revealed that the CT number of rice powder and polyethylene was lower than that of water and the CT number of acrylic, PVC, bone, and aluminum was higher than that of water (Figs 2 and 3). According to these results, it is obvious that all the soft tissue substituted materials (muscle, adipose, and water) with low CT numbers fell closely together at three kVp's. In fact, most of the soft tissue substituted materials (rice powder and polyethylene) had very similar CT number values and bony substituted materials (PVC and bone) were clearly separated from each other with photon beam energy changing, especially at 80 kVp. In addition, it is clear from the results that, for aluminum as a high z material, the CT number difference between the CT scanner and two TPSs decreased with energy compared with bony substituted materials (PVC and bone). The similarity of the CT number values for the substituted materials with low CT numbers might lead to expecting weak dependence of the CT number definition on the photon energy (Fig 2).

Our finding also revealed that the CT numbers of different materials had distinctive behaviors at different energy levels. For the materials with the density of less than water (rice powder, polyethylene, and acrylic), the CT numbers were increased with energy increase from 80 to 130 kVp (Fig 4). This finding was similar to the behaviors of adipose tissues within 10 to 100 keV in Bryant's study [18]. For the materials with the density of higher than water such as PVC, bone, and aluminum, it was decreased with energy increase from 80 to 130 kVp. Although this behavior was in line with Bryant's study for the bone from 20 to 100 keV, for the materials such as blood, lung, brain, and muscle used in the mentioned work, there was a different story in which, from 10 to 20 keV, the CT numbers increased, but afterwards decreased slowly up to 80 keV [19]. Thus, for the materials used as tissue substitute materials, it is better to have the same behavior across the necessary energy range.

The results of this study for rice powder, polyethylene, and acrylic showed a clear trend of increasing CT number with energy in the scanner and two treatment planning systems (Fig 4). For these tissue substitute materials, the CT number values did not significantly change (p-value > 0.05) in the scanner and two treatment planning systems depending on the energy level, except for the polyethylene CT numbers obtained from the MIRS within 110–130 kVp. This finding for polyethylene and acrylic was the same as the results of Cropp's [19] work conducted on the scanner energy ranges of 80 to 140 kVp (Table 3).

We used PVC instead of cancellous bone and aluminum instead of cortical bone. Evaluation of PVC, aluminum, and real cortical bone CT numbers in the scanner and two treatment planning systems (CorePLAN and MIRS) showed no statistically significant difference between the CT number variations of them from the scanner and two treatment planning systems at 80–130 energy levels, except the CT number variations of real cortical bone from the CT scanner at the entire energy levels (80–130 kVp) and PVC from the MIRS at 110–130 kVp (Table 3).

We found no decrease between the CT numbers of aluminum at 80 and 110 kVp. But, after 110 kVp, the CT numbers of aluminum decreased rapidly. However, in both bone and PVC materials, the CT number decreased with all of the energy levels (Fig 4). Thus, our results demonstrated that, as the energy level increased, the CT number decreased in PVC, real bone, and aluminum from 110 to 130 kVp, but the CT number difference was smaller between the scanner and two treatment planning systems for the real bone at this energy level (110 to 130 kVp). These results were in good agreement with those of other studies; in Bryant's study, the direction and magnitude of CT number was descending within 20 to 100 keV for bone [18]. Also, a clear trend of decreasing CT number with kVp from 80 to 140 kVp was observed in Cropp and Zurl's [19, 16] work.

Previous studies have also shown that, for the substances with effective atomic numbers of less than that of water, the CT number increased with energy increasing, while for the materials with the effective atomic number of greater than that of water, the CT number decreased with energy increase [20, 21]. Our finding was also consistent with Okayama et al.'s work, which demonstrated that the CT numbers for several tissues such as the left ventricular cavity, myocardium, and vertebrae decreased as the energy level increased. In contrast, the CT number for the pericardial fat increased with increasing energy level [21].

Based on the physical principles, it can be stated that the CT numbers are derived from the linear attenuation coefficients of materials which depend on the total cross-section for photon interactions. At low energies, the photo-electric interaction dominates and, as the energy rises, the Compton scattering is introduced. The Compton interaction is dominant at the energy levels used in this study (80–130 kVp). We also highlight that the Compton scattering depends on the number of electron per gram, which is higher for the materials with effective low atomic numbers.

In this study, we used different tissue equivalent materials which have been described in the literature [5, 7, 10–22]. According to the results of this study, it is clear that the CT number values of the CT scanner were accurately imported into CorePLAN and MIRS treatment planning systems at each kVp of the scanner for various tissue-equivalent materials (Fig 3).

Comparison of the CT numbers of different tissue equivalent materials between CT scanner and treatment planning systems using statistical paired t-test and percentage differences showed good similarity (Tables 1, 2 and 3), which makes the transferred CT number deviations between the scanner and two treatment planning systems be placed in the recommend tolerance levels [16, 17]. Zurl's work revealed that variations of the CT numbers up to 20% in the HU can result in a mean systematic dose error of 1.5% [16]. In general, the transferred CT numbers of the materials with effective high atomic number increased in CorePLAN treatment planning system, which was not significant. Meanwhile, the MIRS compared with CorePLAN treatment planning systems had a relatively better similarity with the scanner.

The difference in the CT numbers measured in the scanner versus the CT numbers measured from the same images imported into the treatment planning system can be described as follows.

Every CT image is a two-dimensional array of CT values (HU) corresponding to the mean linear attenuation coefficients of the material in each voxel. Linear attenuation coefficients depend on atomic number, electron density, and energy beams of the used in CT scanner. The relationship between relative electron density and CT number in HU is not a straight line, especially for bone substitute materials, in different scanners and depends on scanner performance. Thus, the standard recommendation is that the relationship between electron density and HU be calibrated and verified experimentally for each scanner used for treatment planning [23]. In addition, the results obtained by Yohannes et al. suggested that the tissue equivalent materials used for the measurement or CT number calibration had to have a similar behavior to energy variations [10].

The use of different CT scanning protocols can result in the variations of up to 20% of the HU values [16]. Moreover, in radiotherapy, we need to pay particular attention to the influence of variations in the CT number values on radiation dose calculations in treatment planning systems. Typical Hounsfield unit (HU) conversion into relative electron densities (RED) contributes to dose error by less than 2% [17, 24].

Our results demonstrated that the maximum absolute percentage difference between all of the calculated doses from two treatment planning systems for each photon beam of linac (6 and 15 MV) at three kVp's was less than 1.2% (Tables 4 and 5). Therefore, from the statistical point of view, the transfer of CT number from different kVps of the scanner to treatment planning systems (CorePlan and MIRS) did not have any significant effects on the absorbed dose. But, from the clinical point of view, the influence of variations in the CT number on radiation dose calculations due to different scanning protocols and absence of effective energy of x-ray for accurate definition of CT number values should be considered.

## Conclusion

Accurate dose calculation in radiotherapy not only requires an accurate dose calculation algorithm, but also relies on the accurate calibration of CT numbers for CT-based inhomogeneity corrections. In addition, the CT number may be varied based on the conventional definition of CT number with respect to kVp. So, it is necessary to pay particular attention to the influence of variations in the CT number values on radiation dose calculations in treatment planning systems.

Our results showed that the tissue substitute materials had a different behavior in energy ranges from 80 to 130 kVp. So, it is necessary to consider the energy dependence of substitute materials used for the measurement or calibration of CT number for radiotherapy treatment planning systems. It is expected that, if we use a phantom with materials whose photon beam absorption properties are very similar to those of the tissue, it is possible to accurately carry out the calibration of CT number for radiotherapy treatment planning systems. According to the results of this study, it is clear that the CT number differences at different energies of scanner were smaller with higher kVps (110–130) for the materials with density less than that of water. This issue was the opposite for the materials with the density of higher than water, except for real bone. It is also well known that the scanning of high-density materials with lower kVp can cause an increase in noise and streaking artifacts. So, in the absence of standard photon energy for the definition of CT number, it is recommended to perform CT-scan with high-energy x-ray beams (high kVps).

## Supporting Information

S1 Data Calculated doses of CorePLAN treatment planning system (compressed pdf files). (ZIP)

S2 Data Calculated doses of MIRS treatment planning system (compressed pdf files). (ZIP)
