# Supplementary material for: Energy Dependence of Measured CT Numbers on Substituted Materials Used for CT Number Calibration of Radiotherapy Treatment Planning Systems
Source: PLoS One. 2016 Jul 8;11(7):e0158828. doi: 10.1371/journal.pone.0158828 (PMC4938553; doi:10.1371/journal.pone.0158828)
Supplement: S1 Data — (ZIP) [file pone.0158828.s001.zip › S1_Data/Description of CorePlan output files.pdf]

Detailed Characteristics of output files resulted from the CorePlan TPS

S1 File. Calculated doses of CorePlan treatment planning system on the center of scanned phantom with **real bone at 80 kVp** of scanner using **6 MV** photon beam at the reference field size (10×10 cm^2).

S2 File. Calculated doses of CorePlan treatment planning system on the center of scanned phantom with **real bone at 80 kVp** of scanner using **15 MV** photon beam at the reference field size (10×10 cm^2).

S3 File. Calculated doses of CorePlan treatment planning system on the center of scanned phantom with **real bone at 110 kVp** of scanner using **6 MV** photon beam at the reference field size (10×10 cm^2).

S4 File. Calculated doses of CorePlan treatment planning system on the center of scanned phantom with **real bone at 110 kVp** of scanner using **15 MV** photon beam at the reference field size (10×10 cm^2).

S5 File. Calculated doses of CorePlan treatment planning system on the center of scanned phantom with **real bone at 130 kVp** of scanner using **6 MV** photon beam at the reference field size (10×10 cm^2).

S6 File. Calculated doses of CorePlan treatment planning system on the center of scanned phantom with **real bone at 130 kVp** of scanner using **15 MV** photon beam at the reference field size (10×10 cm^2).

S7 File. Calculated doses of CorePlan treatment planning system on the center of scanned phantom with **PVC at 80 kVp** of scanner using **6 MV** photon beam at the reference field size (10×10 cm^2).

S8 File. Calculated doses of CorePlan treatment planning system on the center of scanned phantom with **PVC at 80 kVp** of scanner using **15 MV** photon beam at the reference field size (10×10 cm^2).

S9 File. Calculated doses of CorePlan treatment planning system on the center of scanned phantom with **PVC at 110 kVp** of scanner using **6 MV** photon beam at the reference field size (10×10 cm^2).

S10 File. Calculated doses of CorePlan treatment planning system on the center of scanned phantom with **PVC at 110 kVp** of scanner using **15 MV** photon beam at the reference field size (10×10 cm^2).

S11 File. Calculated doses of CorePlan treatment planning system on the center of scanned phantom with **PVC at 130 kVp** of scanner using **6 MV** photon beam at the reference field size (10×10 cm^2).

S12 File. Calculated doses of CorePlan treatment planning system on the center of scanned phantom with **PVC at 130 kVp** of scanner using **15 MV** photon beam at the reference field size (10×10 cm^2).

S13 File. Calculated doses of CorePlan treatment planning system on the center of scanned phantom with **water at 80 kVp** of scanner using **6 MV** photon beam at the reference field size (10×10 cm^2).

S14 File. Calculated doses of CorePlan treatment planning system on the center of scanned phantom with **water at 80 kVp** of scanner using **15 MV** photon beam at the reference field size (10×10 cm^2).

S15 File. Calculated doses of CorePlan treatment planning system on the center of scanned phantom with **water at 110 kVp** of scanner using **6 MV** photon beam at the reference field size (10×10 cm^2).

S16 File. Calculated doses of CorePlan treatment planning system on the center of scanned phantom with **water at 110 kVp** of scanner using **15 MV** photon beam at the reference field size (10×10 cm^2).

S17 File. Calculated doses of CorePlan treatment planning system on the center of scanned phantom with **water at 130 kVp** of scanner using **6 MV** photon beam at the reference field size (10×10 cm^2).

S18 File. Calculated doses of CorePlan treatment planning system on the center of scanned phantom with **water at 130 kVp** of scanner using **15 MV** photon beam at the reference field size (10×10 cm^2).

S19 File. Calculated doses of CorePlan treatment planning system on the center of scanned phantom with **aluminum at 80 kVp** of scanner using **6 MV** photon beam at the reference field size (10×10 cm^2).

S20 File. Calculated doses of CorePlan treatment planning system on the center of scanned phantom with **aluminum at 80 kVp** of scanner using **15 MV** photon beam at the reference field size (10×10 cm^2).

S21 File. Calculated doses of CorePlan treatment planning system on the center of scanned phantom with **aluminum at 110 kVp** of scanner using **6 MV** photon beam at the reference field size (10×10 cm^2).

S22 File. Calculated doses of CorePlan treatment planning system on the center of scanned phantom with **aluminum at 110 kVp** of scanner using **15 MV** photon beam at the reference field size (10×10 cm^2).

S23 File. Calculated doses of CorePlan treatment planning system on the center of scanned phantom with **aluminum at 130 kVp** of scanner using **6 MV** photon beam at the reference field size (10×10 cm^2).

S24 File. Calculated doses of CorePlan treatment planning system on the center of scanned phantom with **aluminum at 130 kVp** of scanner using **15 MV** photon beam at the reference field size (10×10 cm^2).
