# Supplementary material for: Energy Dependence of Measured CT Numbers on Substituted Materials Used for CT Number Calibration of Radiotherapy Treatment Planning Systems
Source: PLoS One. 2016 Jul 8;11(7):e0158828. doi: 10.1371/journal.pone.0158828 (PMC4938553; doi:10.1371/journal.pone.0158828)
Supplement: S1 Data — (ZIP) [file pone.0158828.s001.zip › S1_Data/S14_File.pdf]

| | | | |
|---|---|---|---|
| Pat. name: | PHIZIK22_PHIZIK22 | Print Time: | 2014-09-07 09:38:04 |
| Pat. ID: | 132 | Institution: | OMID |
| Study name: | th | Physicist: | |
| Plan name: | Plan 1 | Planner: | |
| | | Comment: | |

## Plan Information

| | |
|---|---|
| Data Set: | DICOM CT, 30 slices<br>512 x 512 pixels, 0.10mm pixel size |
| CT-Density Table Name : | STANDARD |
| Image Patient Position : | HFS |
| Plan Patient Position : | HFS |
| Volume Crop Position : | x1:-24.4, x2:24.3, y1:-24.4, y2:24.3, z1:0.0, z2:29.0 |
| Number of Beams : | PHOTON : 1, ELECTRON : 0 |

| | | | |
|---|---|---|---|
| Pat. name: | PHIZIK22_PHIZIK22 | Print Time: | 2014-09-07 09:38:04 |
| Pat. ID: | 132 | Institution: | OMID |
| Study name: | th | Physicist: | |
| Plan name: | Plan 1 | Planner: | |
| | | Comment: | |

# Beam Summary of 'Presc 1'

Prescription : Presc 1

Prescribe 76.8 cGy(Total) to 100% of PointDose of POI POINT cen for 1 fraction.

Beam weights are proportional to Point Dose.

1 beam(s) are assigned to this prescription.

Actual dose at POI 'POINT cen' is 76.75 cGy.

| | |
|---|---|
| Beam Name | AP |
| Machine | Siemens1 |
| Modality | PHOTON (ETAR) |
| Energy | 15MV |
| Couch Angle | 0.0 degree |
| Gantry Angle | 0.0 degree |
| Collimator Angle | 0.0 degree |
| SSD | 100.1 cm |
| Isocenter | POINT setup (0.4, 18.7, 12.0) |
| Field Size | 10.0 cm * 10.0 cm |
| X1/X2, Y1/Y2 | 5.0/5.0, 5.0/5.0 cm |
| Weight | 100.0 |
| Weight Type | PointDose Proportional |
| Blocked | <Open> |
| Auto Fit Info | <NOT USED> |
| Bolus | <NONE> |
| Tray | <NONE> 1.00 |
| Wedge Name | <OPEN> |
| Wedge Orientation | -- |
| Prescription | Presc 1 |
| Number of Fractions | 1 |
| MU/Fraction | 100.0 |
| --- Add'l Calc. Factors. --- | |
| Reference Point | <Isocenter>, POINT setup |
| Effective Depth | 0.029 |
| OutputFactor | 1.000 |
| Inverse Square | 1.061 |
| Wedge Factor | 1.000 |
| ESF | 1.000 |
| TAR0 | 0.233 |
| Flat. Filter Factor | 0.991 |
| Scatter Factor | 0.092 |
| Tray Factor | 1.000 |
| cGy/MU | 0.341 |

| | | | |
|---|---|---|---|
| Pat. name: | PHIZIK22_PHIZIK22 | Print Time: | 2014-09-07 09:38:04 |
| Pat. ID: | 132 | Institution: | OMID |
| Study name: | th | Physicist: | |
| Plan name: | Plan 1 | Planner: | |
| | | Comment: | |

# POI Dosage

| | TOTAL | AP |
|---|---|---|
| SETUP | 0.0cGy | 0.0cGy |
| (0.00, 0.00, 0.00) | 100% | 0.0% |
| POINT cen | 76.7cGy | 76.8cGy |
| (0.41, 8.81, 12.00) | 100% | 100.0% |
| POINT setup | 17.6cGy | 17.6cGy |
| (0.41, 18.66, 12.00) | 100% | 100.0% |
| Max. Dose | 99.2cGy | 99.2cGy |
| (0.15, 15.85, 12.00) | 100% | 100.0% |
