# Supplementary material for: Energy Dependence of Measured CT Numbers on Substituted Materials Used for CT Number Calibration of Radiotherapy Treatment Planning Systems
Source: PLoS One. 2016 Jul 8;11(7):e0158828. doi: 10.1371/journal.pone.0158828 (PMC4938553; doi:10.1371/journal.pone.0158828)
Supplement: S1 Data — (ZIP) [file pone.0158828.s001.zip › S1_Data/S3_File.pdf]

CorePLAN

Seoul C&J, Inc.
http://www.coreplan.com

Pat. name:	PHIZIK14_PHIZIK14	Print Time:	2014-09-06 17:56:37
Pat. ID:	124	Institution:	OMID
Study name:	th	Physicist:	
Plan name:	Plan 1	Planner:	
		Comment:	

Plan Information

Data Set:	DICOM CT, 30 slices<br>512 x 512 pixels, 0.10mm pixel size
CT-Density Table Name :	STANDARD
Image Patient Position :	HFS
Plan Patient Position :	HFS
Volume Crop Position :	x1:-24.4, x2:24.3, y1:-24.4, y2:24.3, z1:0.0, z2:29.0
Number of Beams :	PHOTON : 1, ELECTRON : 0

CorePLAN

Seoul C&J, Inc.
http://www.coreplan.com

Pat. name:	PHIZIK14_PHIZIK14	Print Time:	2014-09-06 17:56:37
Pat. ID:	124	Institution:	OMID
Study name:	th	Physicist:	
Plan name:	Plan 1	Planner:	
		Comment:	

Beam Summary of 'Presc 1'

Prescription : Presc 1
Prescribe 67.4 cGy(Total) to 100% of PointDose of POI POINT cen for 1 fraction.
Beam weights are proportional to Point Dose.
1 beam(s) are assigned to this prescription.
Actual dose at POI 'POINT cen' is 67.45 cGy.

Beam Name	AP
Machine	Siemens1
Modality	PHOTON (ETAR)
Energy	6MV
Couch Angle	0.0 degree
Gantry Angle	0.0 degree
Collimator Angle	0.0 degree
SSD	100.1 cm
Isocenter	POINT setup (1.1, 13.7, 12.0)
Field Size	10.0 cm * 10.0 cm
X1/X2, Y1/Y2	5.0/5.0, 5.0/5.0 cm
Weight	100.0
Weight Type	PointDose Proportional
Blocked	<Open>
Auto Fit Info	<NOT USED>
Bolus	<NONE>
Tray	<NONE> 1.00
Wedge Name	<OPEN>
Wedge Orientation	--
Prescription	Presc 1
Number of Fractions	1
MU/Fraction	100.0
--- Add'l Calc. Factors. ---	
Reference Point	<Isocenter>, POINT setup
Effective Depth	0.099
OutputFactor	1.000
Inverse Square	1.032
Wedge Factor	1.000
ESF	1.000
TAR0	0.456
Flat. Filter Factor	1.099
Scatter Factor	0.082
Tray Factor	1.000
cGy/MU	0.610

Pat. name:	PHIZIK14_PHIZIK14	Print Time:	2014-09-06 17:56:37
Pat. ID:	124	Institution:	OMID
Study name:	th	Physicist:	
Plan name:	Plan 1	Planner:	
		Comment:	

POI Dosage

	TOTAL	AP
SETUP	0.0cGy	0.0cGy
(0.00, 0.00, 0.00)	100%	0.0%
POINT cen	67.4cGy	67.4cGy
(1.11, 3.80, 12.00)	100%	100.0%
POINT setup	25.8cGy	25.8cGy
(1.11, 13.65, 12.00)	100%	100.0%
Max. Dose	100.6cGy	100.6cGy
(4.15, 11.85, 9.00)	100%	100.0%
