# Supplementary material for: Energy Dependence of Measured CT Numbers on Substituted Materials Used for CT Number Calibration of Radiotherapy Treatment Planning Systems
Source: PLoS One. 2016 Jul 8;11(7):e0158828. doi: 10.1371/journal.pone.0158828 (PMC4938553; doi:10.1371/journal.pone.0158828)
Supplement: S1 Data — (ZIP) [file pone.0158828.s001.zip › S1_Data/S5_File.pdf]

CorePLAN

Pat. name:	PHIZIK15_PHIZIK15	Print Time:	2014-09-07 08:08:06
Pat. ID:	125	Institution:	OMID
Study name:	th	Physicist:	
Plan name:	Plan 1	Planner:	
		Comment:	

Plan Information

Data Set:	DICOM CT, 30 slices<br>512 x 512 pixels, 0.10mm pixel size
CT-Density Table Name :	STANDARD
Image Patient Position :	HFS
Plan Patient Position :	HFS
Volume Crop Position :	x1:-24.4, x2:24.3, y1:-24.4, y2:24.3, z1:0.0, z2:29.0
Number of Beams :	PHOTON : 1, ELECTRON : 0

Pat. name:	PHIZIK15_PHIZIK15	Print Time:	2014-09-07 08:08:06
Pat. ID:	125	Institution:	OMID
Study name:	th	Physicist:	
Plan name:	Plan 1	Planner:	
		Comment:	

Beam Summary of 'Presc 1'

Prescription : Presc 1
Prescribe 67.8 cGy(Total) to 100% of PointDose of POI POINT cen for 1 fraction.
Beam weights are proportional to Point Dose.
1 beam(s) are assigned to this prescription.
Actual dose at POI 'POINT cen' is 67.85 cGy.

Beam Name	AP1
Machine	Siemens1
Modality	PHOTON (ETAR)
Energy	6MV
Couch Angle	0.0 degree
Gantry Angle	0.0 degree
Collimator Angle	0.0 degree
SSD	99.9 cm
Isocenter	POINT setup (1.3, 13.6, 12.0)
Field Size	10.0 cm * 10.0 cm
X1/X2, Y1/Y2	5.0/5.0, 5.0/5.0 cm
Weight	100.0
Weight Type	PointDose Proportional
Blocked	<Open>
Auto Fit Info	<NOT USED>
Bolus	<NONE>
Tray	<NONE> 1.00
Wedge Name	<OPEN>
Wedge Orientation	--
Prescription	Presc 1
Number of Fractions	1
MU/Fraction	100.0
--- Add'l Calc. Factors. ---	
Reference Point	<Isocenter>, POINT setup
Effective Depth	0.121
OutputFactor	1.000
Inverse Square	1.032
Wedge Factor	1.000
ESF	1.000
TAR0	0.461
Flat. Filter Factor	1.099
Scatter Factor	0.082
Tray Factor	1.000
cGy/MU	0.617

Pat. name:	PHIZIK15_PHIZIK15	Print Time:	2014-09-07 08:08:06
Pat. ID:	125	Institution:	OMID
Study name:	th	Physicist:	
Plan name:	Plan 1	Planner:	
		Comment:	

POI Dosage

	TOTAL	AP1
SETUP	0.0cGy	0.0cGy
(0.00, 0.00, 0.00)	100%	0.0%
POINT cen	67.8cGy	67.8cGy
(1.33, 3.78, 12.00)	100%	100.0%
POINT setup	24.4cGy	24.4cGy
(1.33, 13.63, 12.00)	100%	100.0%
Max. Dose	100.4cGy	100.4cGy
(-1.35, 11.85, 9.00)	100%	100.0%
