# Supplementary material for: Energy Dependence of Measured CT Numbers on Substituted Materials Used for CT Number Calibration of Radiotherapy Treatment Planning Systems
Source: PLoS One. 2016 Jul 8;11(7):e0158828. doi: 10.1371/journal.pone.0158828 (PMC4938553; doi:10.1371/journal.pone.0158828)
Supplement: S2 Data — (ZIP) [file pone.0158828.s002.zip › S2_Data/S1_File.pdf]

**NUCLEMED**	**MIRS V5.0.00**
Software Department	User : "Administrator"

Patient	: **Phantom, Mahmodi**	Patient ID	: **1111**	Treat. Date	: **05 Aug 2014**
Case	: **2Bone80**	Case ID	: **2Bone80**	Frame	: **[NONE]**
Diagnostics	: **2Bone80**			Coordinates	: **IEC (mm)**
Position	: **Supine / Patient Head towards Gantry (couch default)**			Origin Name	: **"[No Name]"**

Plan	: **"Plan 1" (Beams:1)**	Type	: **SIMPLE**
Density	: **H-D Curve "Ref"**		
Dose Matrix	: **[Full Anatomy Matrix]**		
Status	: **Calculated**		
Max.Dose	: **102.0 cGy (X=11.5 Y=-213.4 Z=86.2)**		
Norm (Max)	: **102.0 cGy (X=11.5 Y=-213.4 Z=86.2)**		
Global Pr.	: --------		
Approved	: **NOT APPROVED FOR TREATMENT**		

PLANNING DATA REPORT (Page 1 of 1) — 10 Jan 2015 12:55:16 PM

(At reports angles are always shown in Treatment Unit system)

Plan: "Plan 1" / Beam: "AP"

**Treatment Unit**	Name	: **"6MVphoton"**		
	Type	: **LINAC (Photons - 6 MeV)**		**[APPROVED]**
**Isocenter**	Coords	: X=1.2 ; Y=-243.6 ; Z=100.6		
**Collimation**	Name	: **"Default"**	CX (mm)	: **100.0**
	Type	: **Jaw Collimator**	CY (mm)	: **-50.0 / 50.0**
	Shoots	: **1**	Shape	: **Rectangular**
**Modulation**	Type	: **NONE**	Mode	: --------
	Beamlet	: --------	Filter Scheme	: --------
**Incidence**	Arcs	: **1**	Couch	: **0.0°**
	Mode	: **Fixed**	Collimator	: **0.0°**
	SSD	: **1000.0mm**	Gantry	: **0.0°**
**Wedge**	Name	: --------	Insertion	: --------
	Type	: --------	Porcentual	: --------
**Shield**	Applied	: --------	Transmission	: --------
	Type	: --------	Tray	: --------
**Prescription**	Tot.Dose	: **66.7cGy**	Per Fraction	: **66.7cGy**
	Point	: **Point "cal"**	Fractions	: **1**
**Calculation**	Model	: **SI (LR)**	Max.Dose	: **102.0cGy**
	Status	: **Calculated**	Max.Point	: **X=11.5 ; Y=-213.4 ; Z=86.2**
**Irradiation Time**	Fraction	: **100.0 MU**	At Date	: **05 Aug 2014**

Beam Shoot

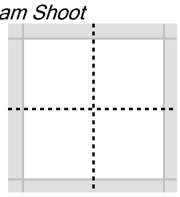

Modulating Filter

(NONE)

Room View

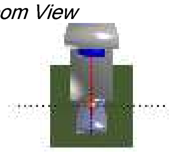

NOTE : Beam is static single shoot so no additional sheets needed to be reported.

Physicist: ____________________ Physician: ____________________
