# Supplementary material for: Energy Dependence of Measured CT Numbers on Substituted Materials Used for CT Number Calibration of Radiotherapy Treatment Planning Systems
Source: PLoS One. 2016 Jul 8;11(7):e0158828. doi: 10.1371/journal.pone.0158828 (PMC4938553; doi:10.1371/journal.pone.0158828)
Supplement: S2 Data — (ZIP) [file pone.0158828.s002.zip › S2_Data/S10_File.pdf]

**NUCLEMED**	**MIRS V5.0.00**
Software Department	User : "Administrator"

Patient	: **Phantim, Mahmodi**	Patient ID	: **2222**	Treat. Date	: **05 Aug 2014**
Case	: **2PVC110**	Case ID	: **2PVC110**	Frame	: **[NONE]**
Diagnostics	: **2PVC110**			Coordinates	: **IEC (mm)**
Position	: **Supine / Patient Head towards Gantry (couch default)**			Origin Name	: **"[No Name]"**

Plan	: **"plan3" (Beams:1)**	Type	: **SIMPLE**
Density	: **Homogeneous [BODY]**		
Dose Matrix	: **[Full Anatomy Matrix]**		
Status	: **Calculated**		
Max.Dose	: **104.2 cGy (X=25.7 Y=-269.8 Z=69.0)**		
Norm (Max)	: **104.2 cGy (X=25.7 Y=-269.8 Z=69.0)**		
Global Pr.	: --------		
Approved	: **NOT APPROVED FOR TREATMENT**		

PLANNING DATA REPORT (Page 1 of 1)	10 Jan 2015 12:02:52 PM

(At reports angles are always shown in Treatment Unit system)

Plan: "plan3" / Beam: "AP"

**Treatment Unit**	Name<br>Type	: **"15MVphoton"**<br>: **LINAC (Photons - 15 MeV)**		**[APPROVED]**
**Isocenter**	Coords	: X=2.6 ; Y=-240.4 ; Z=101.2		
**Collimation**	Name<br>Type<br>Shoots	: **"Default"**<br>: **Jaw Collimator**<br>: **1**	CX (mm)<br>CY (mm)<br>Shape	: **100.0**<br>: **-50.0 / 50.0**<br>: **Rectangular**
**Modulation**	Type<br>Beamlet	: **NONE**<br>: --------	Mode<br>Filter Scheme	: --------<br>: --------
**Incidence**	Arcs<br>Mode<br>SSD	: **1**<br>: **Fixed**<br>: **1000.0mm**	Couch<br>Collimator<br>Gantry	: **0.0°**<br>: **0.0°**<br>: **0.0°**
**Wedge**	Name<br>Type	: --------<br>: --------	Insertion<br>Porcentual	: --------<br>: --------
**Shield**	Applied<br>Type	: --------<br>: --------	Transmission<br>Tray	: --------<br>: --------
**Prescription**	Tot.Dose<br>Point	: **77.4cGy**<br>: **Point "cal"**	Per Fraction<br>Fractions	: **77.4cGy**<br>: **1**
**Calculation**	Model<br>Status	: **SI (LR)**<br>: **Calculated**	Max.Dose<br>Max.Point	: **104.2cGy**<br>: **X=25.7 ; Y=-269.8 ; Z=69.0**
**Irradiation Time**	Fraction	: **100.0 MU**	At Date	: **05 Aug 2014**

Beam Shoot

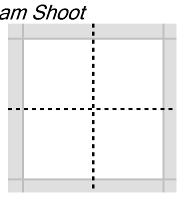

Modulating Filter

(NONE)

Room View

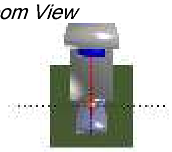

NOTE : Beam is static single shoot so no additional sheets needed to be reported.

Physicist: ....

Physician: ....
