# Supplementary material for: Energy Dependence of Measured CT Numbers on Substituted Materials Used for CT Number Calibration of Radiotherapy Treatment Planning Systems
Source: PLoS One. 2016 Jul 8;11(7):e0158828. doi: 10.1371/journal.pone.0158828 (PMC4938553; doi:10.1371/journal.pone.0158828)
Supplement: S2 Data — (ZIP) [file pone.0158828.s002.zip › S2_Data/S16_File.pdf]

| NUCLEMED | MIRS V5.0.00 |
|---|---|
| Software Department | User : "Administrator" |

| | | | | | |
|---|---|---|---|---|---|
| Patient | : **Phantoom, Mahmoodi** | Patient ID | : **3333** | Treat. Date | : **10 Jan 2015** |
| Case | : **Water110** | Case ID | : **Water110** | Frame | : **[NONE]** |
| Diagnostics | : **Water110** | | | Coordinates | : **IEC (mm)** |
| Position | : **Supine / Patient Head towards Gantry (couch default)** | | | Origin Name | : **"[No Name]"** |

| | | | |
|---|---|---|---|
| Plan | : **"plan3" (Beams:1 )** | Type | : **SIMPLE** |
| Density | : **Homogeneous [BODY]** | | |
| Dose Matrix | : **[Full Anatomy Matrix]** | | |
| Status | : **Calculated** | | |
| Max.Dose | : **104.3 cGy (X=30.1 Y=-166.1 Z=155.6)** | | |
| Norm (Max) | : **104.3 cGy (X=30.1 Y=-166.1 Z=155.6)** | | |
| Global Pr. | : -------- | | |
| Approved | : **NOT APPROVED FOR TREATMENT** | | |



*(At reports angles are always shown in Treatment Unit system)*

## Plan: "plan3" / Beam: "AP"

| | | | | |
|---|---|---|---|---|
| **Treatment Unit** | Name<br>Type | : **"15MVphoton"**<br>: **LINAC (Photons - 15 MeV)** | | **[ APPROVED ]** |
| **Isocenter** | Coords | : X=7.3 ; Y=-190.8 ; Z=187.3 | | |
| **Collimation** | Name<br>Type<br>Shoots | : **"Default"**<br>: **Jaw Collimator**<br>: **1** | CX (mm)<br>CY (mm)<br>Shape | : **100.0**<br>: **-50.0 / 50.0**<br>: **Rectangular** |
| **Modulation** | Type<br>Beamlet | : **NONE**<br>: -------- | Mode<br>Filter Scheme | : --------<br>: -------- |
| **Incidence** | Arcs<br>Mode<br>SSD | : **1**<br>: **Fixed**<br>: **1000.0mm** | Couch<br>Collimator<br>Gantry | : **0.0°**<br>: **0.0°**<br>: **0.0°** |
| **Wedge** | Name<br>Type | : --------<br>: -------- | Insertion<br>Porcentual | : --------<br>: -------- |
| **Shield** | Applied<br>Type | : --------<br>: -------- | Transmission<br>Tray | : --------<br>: -------- |
| **Prescription** | Tot.Dose<br>Point | : **77.6cGy**<br>: **Point "cal"** | Per Fraction<br>Fractions | : **77.6cGy**<br>: **1** |
| **Calculation** | Model<br>Status | : **SI (LR)**<br>: **Calculated** | Max.Dose<br>Max.Point | : **104.3cGy**<br>: **X=30.1 ; Y=-166.1 ; Z=155.6** |
| **Irradiation Time** | Fraction | : **100.0 MU** | At Date | : **10 Jan 2015** |

*Beam Shoot*

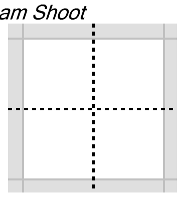

*Modulating Filter*

(NONE)

*Room View*

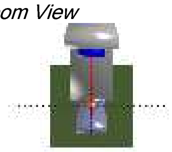

**NOTE : Beam is static single shoot so no additional sheets needed to be reported.**

Physicist: ____________________ Physician: ____________________
