# Supplementary material for: Energy Dependence of Measured CT Numbers on Substituted Materials Used for CT Number Calibration of Radiotherapy Treatment Planning Systems
Source: PLoS One. 2016 Jul 8;11(7):e0158828. doi: 10.1371/journal.pone.0158828 (PMC4938553; doi:10.1371/journal.pone.0158828)
Supplement: S2 Data — (ZIP) [file pone.0158828.s002.zip › S2_Data/S5_File.pdf]

Patient	: **Phantom, Mahmodi**	Patient ID	: **1111**	Treat. Date	: **11 Aug 2014**
Case	: **2Bone130**	Case ID	: **2Bone130**	Frame	: **[NONE]**
Diagnostics	: **2Bone130**			Coordinates	: **IEC (mm)**
Position	: **Supine / Patient Head towards Gantry (couch default)**			Origin Name	: **"[No Name]"**

Plan	: **"Plan 1" (Beams:1)**	Type	: **SIMPLE**
Density	: **H-D Curve "Ref"**		
Dose Matrix	: **[Full Anatomy Matrix]**		
Status	: **Calculated**		
Max.Dose	: **101.6 cGy (X=8.6 Y=-267.0 Z=88.1)**		
Norm (Max)	: **101.6 cGy (X=8.6 Y=-267.0 Z=88.1)**		
Global Pr.	: --------		
Approved	: **NOT APPROVED FOR TREATMENT**		

PLANNING DATA REPORT (Page 1 of 1)

10 Jan 2015 1:43:41 PM

(At reports angles are always shown in Treatment Unit system)

Plan: "Plan 1" / Beam: "AP"

**Treatment Unit**	Name	: **"6MVphoton"**		
	Type	: **LINAC (Photons - 6 MeV)**	**[APPROVED]**	
**Isocenter**	Coords	: X=2.4 ; Y=-241.2 ; Z=100.9		
**Collimation**	Name	: **"Default"**	CX (mm)	: **100.0**
	Type	: **Jaw Collimator**	CY (mm)	: **-50.0 / 50.0**
	Shoots	: **1**	Shape	: **Rectangular**
**Modulation**	Type	: **NONE**	Mode	: --------
	Beamlet	: --------	Filter Scheme	: --------
**Incidence**	Arcs	: **1**	Couch	: **0.0°**
	Mode	: **Fixed**	Collimator	: **0.0°**
	SSD	: **1000.0mm**	Gantry	: **0.0°**
**Wedge**	Name	: --------	Insertion	: --------
	Type	: --------	Porcentual	: --------
**Shield**	Applied	: --------	Transmission	: --------
	Type	: --------	Tray	: --------
**Prescription**	Tot.Dose	: **66.3cGy**	Per Fraction	: **66.3cGy**
	Point	: **Point "cal"**	Fractions	: **1**
**Calculation**	Model	: **SI (LR)**	Max.Dose	: **101.6cGy**
	Status	: **Calculated**	Max.Point	: **X=8.6 ; Y=-267.0 ; Z=88.1**
**Irradiation Time**	Fraction	: **100.0 MU**	At Date	: **11 Aug 2014**

Beam Shoot

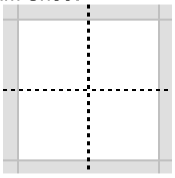

Modulating Filter

(NONE)

Room View

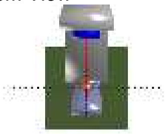

NOTE : Beam is static single shoot so no additional sheets needed to be reported.

Physicist: ____________________ Physician: ____________________
