# Supplementary material for: Energy Dependence of Measured CT Numbers on Substituted Materials Used for CT Number Calibration of Radiotherapy Treatment Planning Systems
Source: PLoS One. 2016 Jul 8;11(7):e0158828. doi: 10.1371/journal.pone.0158828 (PMC4938553; doi:10.1371/journal.pone.0158828)
Supplement: S2 Data — (ZIP) [file pone.0158828.s002.zip › S2_Data/S8_File.pdf]

NUCLEMED	MIRS V5.0.00
Software Department	User : "Administrator"

Patient	: Phantim, Mahmodi	Patient ID	: 2222	Treat. Date	: 05 Aug 2014
Case	: 2PVC80	Case ID	: 2PVC80	Frame	: [NONE]
Diagnostics	: 2PVC80			Coordinates	: IEC (mm)
Position	: Supine / Patient Head towards Gantry (couch default)			Origin Name	: "[No Name]"

Plan	: "plan3" (Beams:1)	Type	: SIMPLE
Density	: Homogeneous [BODY]		
Dose Matrix	: [Full Anatomy Matrix]		
Status	: Calculated		
Max.Dose	: 104.2 cGy (X=-20.2 Y=-269.8 Z=69.2)		
Norm (Max)	: 104.2 cGy (X=-20.2 Y=-269.8 Z=69.2)		
Global Pr.	: --------		
Approved	: NOT APPROVED FOR TREATMENT		

PLANNING DATA REPORT (Page 1 of 1) — 10 Jan 2015 11:34:52 AM

(At reports angles are always shown in Treatment Unit system)

Plan: "plan3" / Beam: "AP"

**Treatment Unit**	Name	: "15MVphoton"		
	Type	: LINAC (Photons - 15 MeV)	[APPROVED]	
**Isocenter**	Coords	: X=2.6 ; Y=-239.8 ; Z=101.2		
**Collimation**	Name	: "Default"	CX (mm)	: 100.0
	Type	: Jaw Collimator	CY (mm)	: -50.0 / 50.0
	Shoots	: 1	Shape	: Rectangular
**Modulation**	Type	: NONE	Mode	: --------
	Beamlet	: --------	Filter Scheme	: --------
**Incidence**	Arcs	: 1	Couch	: 0.0°
	Mode	: Fixed	Collimator	: 0.0°
	SSD	: 1000.0mm	Gantry	: 0.0°
**Wedge**	Name	: --------	Insertion	: --------
	Type	: --------	Porcentual	: --------
**Shield**	Applied	: --------	Transmission	: --------
	Type	: --------	Tray	: --------
**Prescription**	Tot.Dose	: 77.5cGy	Per Fraction	: 77.5cGy
	Point	: Point "cal"	Fractions	: 1
**Calculation**	Model	: SI (LR)	Max.Dose	: 104.2cGy
	Status	: Calculated	Max.Point	: X=-20.2 ; Y=-269.8 ; Z=69.2
**Irradiation Time**	Fraction	: 100.0 MU	At Date	: 05 Aug 2014

Beam Shoot

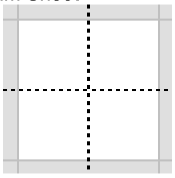

Modulating Filter

(NONE)

Room View

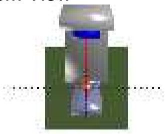

NOTE : Beam is static single shoot so no additional sheets needed to be reported.

Physicist: ____________ Physician: ____________
